# Triaging Patient Complaints: Monte Carlo Cross-Validation of Six Machine Learning Classifiers

**DOI:** 10.2196/medinform.7140

**Published:** 2017-07-31

**Authors:** Adel Elmessiry, William O Cooper, Thomas F Catron, Jan Karrass, Zhe Zhang, Munindar P Singh

**Affiliations:** ^1^ North Carolina State University Department of Computer Science Raleigh, NC United States; ^2^ Vanderbilt University Medical Center Nashville, TN United States; ^3^ IBM Research Triangle Park, NC United States

**Keywords:** natural language processing, NLP, machine learning, patient complaints

## Abstract

**Background:**

Unsolicited patient complaints can be a useful service recovery tool for health care organizations. Some patient complaints contain information that may necessitate further action on the part of the health care organization and/or the health care professional. Current approaches depend on the manual processing of patient complaints, which can be costly, slow, and challenging in terms of scalability.

**Objective:**

The aim of this study was to evaluate automatic patient triage, which can potentially improve response time and provide much-needed scale, thereby enhancing opportunities to encourage physicians to self-regulate.

**Methods:**

We implemented a comparison of several well-known machine learning classifiers to detect whether a complaint was associated with a physician or his/her medical practice. We compared these classifiers using a real-life dataset containing 14,335 patient complaints associated with 768 physicians that was extracted from patient complaints collected by the Patient Advocacy Reporting System developed at Vanderbilt University and associated institutions. We conducted a 10-splits Monte Carlo cross-validation to validate our results.

**Results:**

We achieved an accuracy of 82% and F-score of 81% in correctly classifying patient complaints with sensitivity and specificity of 0.76 and 0.87, respectively.

**Conclusions:**

We demonstrate that natural language processing methods based on modeling patient complaint text can be effective in identifying those patient complaints requiring physician action.

## Introduction

Patient complaints are an important source of information for health care organizations for improving the patient experience. Patients are uniquely positioned to make observations about the care they receive, particularly when they complain when health care professionals or organizations fail to meet their expectations. When patients and family members share their observations, organizations can engage in service recovery, the process of “making right” what went wrong for patients and families [1]. Most patient complaints can be resolved at the point of service and require no additional action. However, when a patient expresses dissatisfaction about some aspect of a physician’s practice, it is important to share that information with the physician so that he or she can reflect on the situation and potentially develop strategies for preventing the recurrence of the events that engendered the initial dissatisfaction [2]. When patterns develop, reviewing patient complaints offers insight into sources of potential continuing patient dissatisfaction that can be addressed by the medical professional and/or the organization. Some patient and family complaints require immediate response by the organization and/or the health care professional because review and response is required by law, regulation, or policy [3] (eg, sexual boundary violation, drug or alcohol impairment in the workplace).

Many health care organizations receive thousands of unsolicited patient complaints a year [4-6]. Manual review of these complaints by trained coders has been shown to be reliable and valid [7], but it is time consuming and may occur some weeks or months after the complaint is received. In addition, scalability of human coding presents logistical and time challenges. Thus, there is a need to triage patient complaints to identify complaints that should be shared with the involved physician(s). We describe a study in which we implemented several well-known machine learning classifiers to optimally detect patient complaints about physicians’ practices using data from the Patient Advocacy Reporting System (PARS), a national program that draws data from multiple hospitals’ patient complaint reporting systems to identify professionalism concerns and malpractice risk among health care professionals [7].

### Problem, Challenges, and Approach in Brief

We posit that complaint text can be used to discern the relevance of a complaint to a physician and thus correctly and efficiently identify which complaints should be shared. The goal of this study is to determine whether a given patient complaint can be shared with the physician with the same level of accuracy that is achieved with existing manual approaches.

Our problem is challenging due to the following factors:

1. Physician practice-related complaints are not always easy to characterize. The vocabulary used to describe physician-related complaints overlaps with that used for other types of complaints (eg, billing), partly because of the common effect of the medical setting.
2. Achieving optimal accuracy is a delicate balance. Failing to detect a physician-related complaint results in continued patient dissatisfaction that could have been successfully addressed. On the other hand, false positive instances, where unrelated complaints are shared with physicians, would result in wasted time and effort.
3. Text for a single complaint may gather multiple perspectives, including the patient and the patient’s family, friends, and care providers. These parties have different and possibly conflicting objectives. In most cases, patient advocates record patient complaints in the system using patient words without rewording or paraphrasing. The advocate may insert their impression, such as “the patient was angry” or “the patient was shouting.” Patient advocates may add subsequent actions and responses to the patient complaint. In other cases, the complaint process begins when a patient writes a letter to the medical center, in which case the advocates would take snippets from the actual letter. There can be paraphrasing depending on the organization and the individual advocate.

Our approach involves extracting common features from physician-related patient complaints that have already been correctly classified as such by a team of human coders. Those common features are then applied to a second group of patient complaints in order to classify them as either physician-related or non-physician-related complaints.

For our comparisons, we (1) implement a framework that employs six well-known classifiers and (2) experiment with two methods of feature extraction from complaint text.

### Related Work

The bulk of the textual artifacts in health care can be found in two main sources: clinical and nonclinical. Clinical textual artifacts are largely entries in the medical chart, comments on the case, or physician notes. Medical chart notes tend to be consciously made and well structured, whereas case comments and physician notes focus on treatment (including diagnoses) of the patient. Nonclinical textual artifacts include unsolicited patient feedback and often revolve around complaints. The text is variable, may contain abbreviations, and may extend beyond the actual treatment or diagnosis.

Previous research has focused on clinical textual artifacts [8]. Recent research demonstrates the possibility to apply natural language processing (NLP) on electronic medical records to identify postoperative complications [9]. Bejan and Denny [10] showed how to identify treatment relationships in clinical text using a supervised learning system that is able to predict whether or not a whether or not a treatment relation exists between any two medical concepts mentioned in the clinical notes exists between any two medical concepts mentioned in the clinical notes.

Cui et al [11] explored a large number of consumer health questions. For each question, they selected a smaller set of the most relevant concepts adopting the idea of the term frequency-inverse document frequency (TF-IDF) metric. Instead of computing the TF-IDF based on the terms, they used concept unique identifiers. Their results indicate that we can infer more information from patient comments than commonly thought. However, questions are short and limited, whereas patient complaints are rich and elaborate.

Sakai et al [12] concluded that how risk assessment and classification is configured is often a decisive intervention in the reorganization of the work process in emergency services. They demonstrated the textual analysis of feedback provided by nurses can expose the sentiment and feelings of the emergency workers and help improve the outcomes.

Temporal information in discharge summaries has been successfully used [13] to classify encounters, enabling the placement of data within the structure to provide a foundational representation on which further reasoning, including the addition of domain knowledge, can be accomplished.

Additional research [14] extended the clinical Text Analysis and Knowledge Extraction System (cTAKES) with a simplified feature extraction, and the development of both rule and machine learning-based document classifiers. The resulting system, the Yale cTAKES Extensions (YTEX), can help classify radiology reports containing findings suggestive of hepatic decompensation. A recent systematic literature review of 85 articles focusing on the secondary use of structured patient records showed that electronic health record data structuring methods are often described ambiguously and may lack clear definition as such [15].

### Complaints

For the objective of this research, we group complaints into two main categories, as described subsequently.

#### Complaints Involving a Physician

These are complaints can be inferred to be, and are, associated with a physician’s practice:

Dr XXX seemed more concerned with getting to her next patient than to listening to what I had to say.

After the procedure she asked Dr XXX if he would be speaking with her dad. He said no, he tells the family and they can tell the pt [patient]. The daughter does not feel it was her place to discuss with her dad that he has terminal cancer..

The patient asked the doctor to give her an x-ray, but he refused. Two days later, the patient went to the emergency room and an x-ray showed that her arm was broken.

Obviously, Dr XXX did not review his medical chart

Dr XXX rushed through the appointment.

I arrived early for my appointment but had to wait almost 2 hours to be seen. This happens every time I see Dr XXX.

#### Complaints Not Involving a Physician or His/Her Practice

These are complaints that concern billing or requesting information (or are not a complaint at all). They normally do not require medical escalation and can be typically handled by the staff:

Patient has contacted our office multiple times to get assistance with getting her CPAP machine repaired. She stated that we had not given her home health company the needed information.

The ER triage RN “treated her husband like garbage.” [The inpatient] RN “the attitude queen would not call the doctor for a sleeping medication” and that the service coordinator was “rude and stated the manager of the unit refused to speak to her.”

Mrs X was scheduled for an appointment in the North office on October 20. She was told that her appointment would be in the East location. Mrs X’s son traveled a couple of hours to bring his mother to her appointment. When they arrived for her appointment, there was no one in the East office so they left and went home.

She sat in the ER last night from 7:45 pm to 8:20 pm without being triaged. Patient states she has asthma and she was having a severe allergic reaction. Patient states a young male RN told her she would be seen next but the other triage RN called seven people before her.

### Human Coders

Each unsolicited patient complaint report in our dataset had previously been reviewed by a trained research assistant and identified as either containing a complaint about a physician or not. These 15 research assistants received extensive training on the classification protocol and met internally developed reliability standards [7]. The standard of reliability was an alpha of 0.80 or higher [16,17]. The interrater agreement reliability between pairs of research assistants ranged from 0.70 to 0.95, with a median alpha of 0.86. The intercoder agreement was high due to the extensive training the coders underwent on the PARS classification.

## Methods

No single term or attribute signifies whether or not a patient complaint involves a physician and/or his or her medical practice. Therefore, we approached the problem by clustering text into one of two clusters. Documents are commonly represented as a sparse vector over the entire feature set consisting of all distinct terms over all documents. Two major drawbacks are (1) high dimensionality (ie, a large number of features) and (2) feature sparsity (ie, features appearing in only a few documents) [18].

Accordingly, we implemented a framework that consisted of the following steps: (1) preprocessed the documents to remove common stop words and numbers and to perform stemming (eg, the stem “argu” would replace “argue,” “argued,” “argues,” “arguing,” and “argus”); (2) ran Monte Carlo cross-validation [19] using 10 splits and for each we randomly sampled an 80% training and 20% testing dataset from our corpus (approximately 11,468 training complaints and 2867 testing complaints), extracted features through generating sparse representation of the documents based on TF or TF-IDF, reduced features by removing sparse terms, and trained a model to predict the labels; (3) computed the mean accuracy, sensitivity, and specificity for each classifier; and (4) selected the best-performing classifier.

### Feature Extraction

The first step was to map patient complaints to a set of representative features. Wilcox and Hripcsak [20] showed that domain knowledge representation can vary between task-specific and representation-specific knowledge. Medical knowledge is specific to the conditions being identified and essential for clinical report classification. As in our case, Wilcox and Hripcsak emphasized attribute or feature extraction. Generating medically relevant features requires an understanding of the medical report or the underlying meaning of the text. Our approach associates medical relevance with feature relevance to the document.

We compared two methods for feature extraction, namely TF and TF-IDF. TF-IDF seeks to emphasize the importance of a word to a document in a collection or corpus [21]. In information classification and retrieval, TF-IDF is widely used [22]. The idea is simply to multiply the TF with IDF computed with respect to the entire corpus as shown in Equation 1:

(1) TF-IDF(t)=tf(t,d)×log(N/n
_t_)

where *tf* (*t,d*) counts the frequency by which term *t* appears in document *d*, *N* is the total number of documents in the corpus, and *n*_t_ is the number of documents in which the term *t* appears.

The idea of incorporating IDF is to reduce the weight on words that occur frequently in each document, but are not sufficiently selective. For example, the words “her” and “nurse” would occur too commonly in patient complaints to be useful for retrieval or selection.

We adopted TF-IDF for feature extraction as follows: (1) generated a vocabulary of unique terms, (2) generated term frequency per document, (3) generated inverse document weight per term, and (4) replaced the frequency with the TF-IDF weights using Equation 1. The result was a sparse vector representation of the document.

### Feature Reduction

Feature reduction aims at reducing the number of features while maintaining the underlying meaning of the document. A smaller number of representative features can maintain a comparable level of prediction performance while reducing noise and unnecessary processing. Both TF and TF-IDF generated a large number of features, the majority of which are not relevant in predicting whether a complaint involves a physician. To reduce the number of features, we removed sparse features. We applied a similar definition of the term sparsity described in Saif et al [23], which can be defined as the ratio of the number of documents in which this term appears and the total number of documents in the corpus, as shown in Equation 2:

(2) Sparsity=n
_t_/N

where *n*_t_ is the number of documents in which the term *t* appears and *N* is the total number of documents in the corpus. A term with 0.90 sparsity means the term appears in at least 90% of the documents, whereas a term with 0.99 sparsity appears in at least 99% of the documents.

We repeated the Monte Carlo cross-validation training and prediction while varying the sparsity from 0.90 to 0.99 to assess the minimum number of features to select and still maintain the desired prediction performance. A reduced number of representative features is desirable because it reduces the size of the model while maintaining the accuracy. The following example shows some selected word stem features organized into four groups for illustration purposes: (1) financial account, charge, close, bill, and call; (2) medical cardiac, cardiology, complications, injury, and coronary; (3) facility center, clinic, access, action, and assist; and (4) care complaint, concern, attach, and care.

### Classifier Selection

The final step was to assess the best classifier to employ for our problem. Due to the special nature of the problem, selecting a classifier prospectively was difficult. We implemented a supervised learning framework to capture the relation between patient text and the resultant physician action. The models then could detect whether the complaint was related to a physician’s practice. Our framework supported six well-known classifiers. We used RTextTools [24] as the library to implement the classifiers shown in Table 1. After experimenting with these classifiers on the same dataset, we selected the best overall performing classifier.

### Evaluation

We divided the dataset into a training and a testing dataset. We used one of the six classifiers to train a model over the mapped dataset. We then used the testing dataset to validate the accuracy of our classifiers. Accuracy is defined by Equation 3:

(3) Accuracy=(true positives+true negatives)/(true positives+false positives+true negatives+false negatives)

Sensitivity captures how many patients with a condition are detected (ie, the avoidance of false negatives) as in Equation 4:

(4) Sensitivity=true positives/(true positives+false negatives)

Specificity captures how many patients without a condition are not detected (ie, the avoidance of false positives) as shown in Equation 5:

(5) Specificity=true negatives/(true negatives+false positives)

The F-score captures how accurate the test was. It is computed using both the precision and the recall as shown in Equation 6:

(6) F-score=2×(precision×recall) /(precision+recall)

### Institutional Review Board Approval

This research was reviewed and approved by the Vanderbilt Medical Center Institutional Review Board and the North Carolina State University Institutional Review Board.

## Results

**Figure 1:**
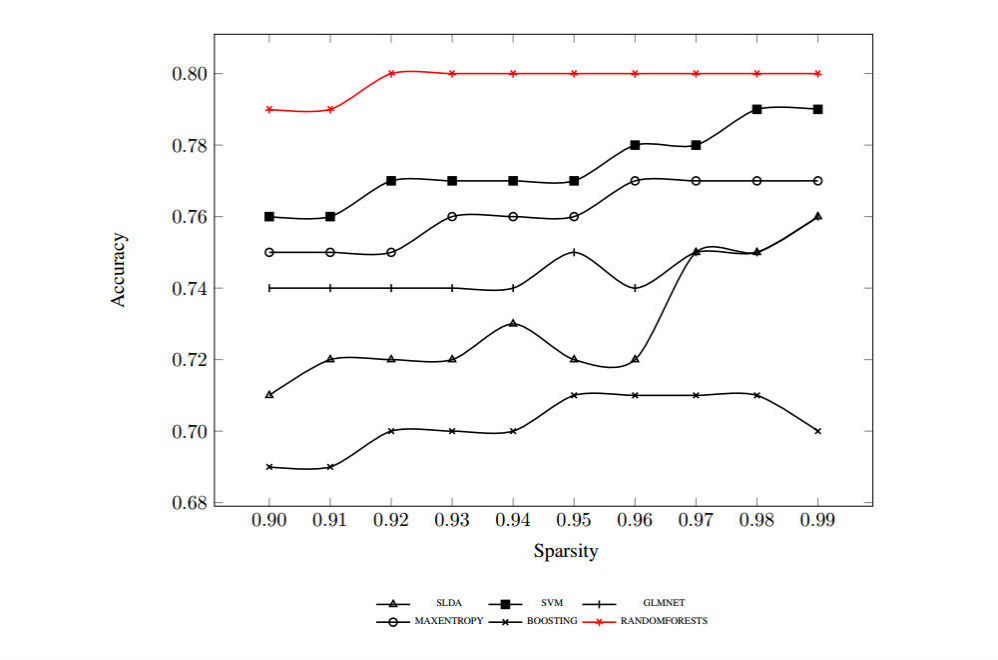
Term frequency-generated features using 10-splits Monte Carlo cross-validation accuracy.

We first report our full 10-splits results for each classifier's predictions. Figure 1 shows the results obtained using TF-extracted features. We experimented with changing the sparsity from 0.90 to 0.99 to reduce the number of selected features. The prediction accuracy either slightly improved or remained steady with the reduced number of features except in the random forests case, where the accuracy peaked and dropped slightly at the end of the range.

**Figure 2:**
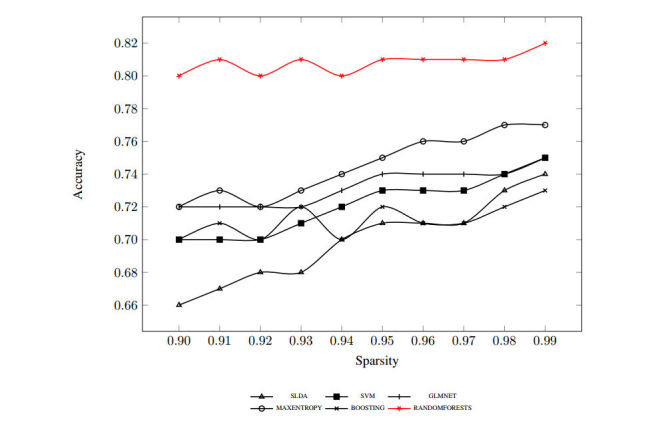
Term frequency-inverse document frequency-generated features using 10-splits Monte Carlo cross-validation accuracy.

The case is a bit different with results obtained using TF-IDF-extracted features, as shown in Figure 2. The prediction of all classifiers improved notably (from 2.5% in the case of random forests to 12.1% in the case of SLDA) because we reduced the number of selected features. The gap between the best-performing classifier using TF-IDF and the rest of the classifiers was more pronounced as well. Because results were generally better at higher sparsity, we reported the detailed results at sparsity of 0.99 with accuracy, sensitivity, and specificity for both TF and TF-IDF in Table 2 as well as the harmonic mean (F-score) over each of the six classifiers we implemented.

**Table 1:** Implemented classifiers.

Classifier	Description
Scaled linear discriminant analysis (SLDA)	Expresses one dependent variable as a linear combination of other variables. SLDA is similar to ANOVA, but with the difference that SLDA assumes continuous independent variables and categorical dependent labels. SLDA is widely used in image and pattern recognition [25].
Support vector machines (SVM)	Divides the dataset via a set of hyperplanes during the learning phase and maps new data to fall into one of the hyperplanes. SVM has been used for text classification [26].
Glmnet	An implementation of the Lasso and elastic-net regularized generalized linear models, Glmnet is popular for domains with large databases [27].
Max entropy	A probabilistic classifier that selects the model with maximum entropy from among a set of models and uses it to classify data [28].
Boosting	Aggregates a set of weak learners (classifiers that perform slightly better than random) to create a strong learner by weighting them appropriately [29].
Random forests	An ensemble learning method, similar to boosting, that learns and combines many decision trees and subsequently selects the best performing from among multiple learning algorithms to improve predictions.

**Table 2:** Classifiers term frequency (TF) versus term frequency-inverse document frequency (TF-IDF) accuracy, sensitivity, specificity, and F-score using 10-splits Monte Carlo cross-validation at 0.99 sparsity.

Classifier	TF				TF-IDF			
	Accuracy	Sensitivity	Specificity	F-score	Accuracy	Sensitivity	Specificity	F-score
SLDA	0.76	0.72	0.80	0.76	0.74	0.66	0.83	0.74
SVM	0.79	0.71	0.86	0.78	0.75	0.67	0.82	0.74
Glmnet	0.76	0.71	0.81	0.75	0.76	0.64	0.86	0.73
Max entropy	0.77	0.71	0.83	0.76	0.77	0.69	0.84	0.76
Boosting	0.70	0.85	0.55	0.67	0.73	0.82	0.64	0.72
Random forests	0.80	0.74	0.87	0.80	0.82	0.76	0.87	0.81

## Discussion

The results of this study indicate that a machine learning approach can be effective in identifying patient complaints that involve physicians. It is interesting that using term sparsity to reduce the feature set provides robust improvement until we arrive at a point where the terms are too few to provide any meaningful discrimination between the labels and, thus, the prediction accuracy falls. Adding IDF adjusts the weights assigned by TF in TF-IDF, which helps remove features that do not contribute significant information. Although common terms would be more prone to appear in the TF less sparse terms, the TF-IDF would have removed those terms before we get to this point. Our results are consistent with prior research (eg, Liu et al [30] and Cho and Lee [31]), showing improved results with a reduced (and hence a more representative) set of features. The insight here is that although reducing the number of features leads to better prediction performance, knowing which features to keep plays a significant role as well.

Our specific findings are that the best-performing classifier was random forests with 82% accuracy and 81% F-score using TF-IDF for feature generation, followed by the SVM classifier, which achieved 79% accuracy using the simpler TF for feature generation. Adopting our automated approach would lead to the identification of patient complaints that should be shared with a physician much faster than any manual approach and thereby encourage thoughtful review and potential improvements.

### Error Analysis

Error analysis is a critical step to understanding the failure mode of the classifiers [32]. We attempted to understand the general trends underpinning the classifier error. In Table 3, we show the percentage of total false prediction, positives and negatives, versus the number of classifiers that shared the error prediction.

**Table 3:** Classifier error analysis (n=3010).

Number of classifiers sharing an error prediction	% of errors
6	17.97
5	43.99
4	1.99
3	1.00
2	1.00
1	33.99

We note that in 61.96% (1865/3010) of cases, at least five of the six used classifiers shared the erroneous prediction. In 98.80% (2974/3010) of those cases, the classifiers predicted that the complaint required physician action, although it did not.

We wanted to understand why the classifiers were confused in this specific manner. In analyzing the complaints, a pattern emerged. The complaints mainly shared a few topics: patient falling, medical records, or billing issue. The terms used in those complaints contain a mix of both cases because a physician may be involved or mentioned in those cases and the complaint topic does not require physician action. The insight we draw from our error analysis is that although TF-IDF provides a good approach for weighting the features, it is not sensitive enough to distinguish mixed cases. Potential methods for alleviating the errors that appear in patient falling, medical records, and billing issues would potentially include using dependency-based features [33,34] to capture contextual information or a health care-specific lexicon.

### Limitations

Modeling the content of patient complaints is a challenging problem. We limited feature extraction to TF and TF-IDF, which although generating robust results, still leaves unanswered the question of whether more useful data could yet be extracted. Using TF-IDF does not always work well. For example, the term “doctor” was very frequent and is an important feature, although it was not determined to be important using TF-IDF due to the prevalence of the term in the medical domain. TF-IDF can easily confuse such terms with more noisy terms as illustrated with the term “her.”

Exploring more advanced NLP methods to dive into the underlying language structure and reduce the noise would represent a potential future line of inquiry. Although 82% accuracy and 81% F-score is a promising start in regards to our specific problem, extracting better features may help improve the accuracy. Another limitation of our work is our focus on the binary classification we have used. Patient complaints involving physicians’ practices are not all the same; rather, some may be treatment concerns, environmental issues, physician behavioral issues, or competency questions. It would be interesting to expand our scope to address those issues.

### Future Directions

A future direction is to extend methods outlined by Tausczik and Pennebaker [35] and Zhang and Singh [36] to build a lexicon specific to health care complaints, which could yield superior metrics such as accuracy. Another interesting direction is to evaluate the influence of geography; specifically, do patients from different locations express themselves differently and do their differences in phrasing affect the underlying meaning?

## Abbreviations

NLP: natural language processing
PARS: Patient Advocacy Reporting System
SLDA: scaled linear discriminant analysis
SVM: support vector machines
TF: term frequency
TF-IDF: term frequency-inverse document frequency
